# D-Shaped POF Sensors for Refractive Index Sensing—The Importance of Surface Roughness

**DOI:** 10.3390/s19112476

**Published:** 2019-05-30

**Authors:** Filipa Sequeira, Nunzio Cennamo, Alisa Rudnitskaya, Rogério Nogueira, Luigi Zeni, Lúcia Bilro

**Affiliations:** 1 Instituto de Telecomunicações, 3810-193 Aveiro, Portugal; rnogueira@av.it.pt (R.N.); lucia.bilro@av.it.pt (L.B.); 2 Department of Physics, University of Aveiro, 3810-193 Aveiro, Portugal; 3 CESAM, University of Aveiro, 3810-193 Aveiro, Portugal; alisa@ua.pt; 4 Department of Engineering, University of Campania Luigi Vanvitelli, 81031 Aversa, Italy; nunzio.cennamo@unicampania.it (N.C.); luigi.zeni@unicampania.it (L.Z.); 5 Department of Chemistry, University of Aveiro, 3810-193 Aveiro, Portugal

**Keywords:** optical fiber sensors (OFS), plastic optical fibers (POF), D-shaped POF sensors, low-cost sensors, intensity modulation, refractive index (RI) sensing, surface roughness

## Abstract

In this study the influence of the surface roughness on the transmission capacities of D-shaped plastic optical fibers (POFs) and sensors performance was investigated. Five D-shaped POF sensors were produced and characterized for refractive index sensing between 1.33 and 1.41. The sensors were characterized using a low-cost optical sensing system based on the variation of the transmitted light though the POF with refractive index changes (RI). Higher surface roughness increases the scattering losses through the POF and influences the sensors’ performance; therefore, a balance must be attained. Generally, the best performance was achieved when the sensing region was polished with P600 sandpaper as a final polishing step. Polishing with sandpapers of lower grit size resulted in lower scattering, higher linearity of the sensor response and generally lower performance for RI sensing. A sensor resolution of 10^−3^–10^−4^ RIU, dependent on the value of the external refractive index, was obtained through simple and low-cost manufacturing procedures. The obtained results show the importance of surface roughness in the development of POF sensors which can be used in several applications, such as for water quality assessment.

## 1. Introduction

Nowadays, the assessment of water quality is of utmost importance for sustainable living and even for the survival of the human species and biodiversity. Water sources, namely, seas, lakes, and subterranean and superficial waters, are commonly contaminated with chemical species from various sources, from industry, agriculture with the excessive use of herbicides and fertilizers, landfill deposition of contaminated garbage, and even health centers, universities, technological institutes, and households. Despite constant technological developments, Waste Water Treatment Plants still do not have the capability of detection and removal of all the contaminants that are present in the waste waters and which, unfortunately, end up in water bodies [1,2,3]. The development of sensors for water quality assessment which would allow remote and on-site measurements can promote a new age in environmental monitoring. Nowadays, there is still the need for sample collection and analysis in a certificated laboratory with the use of high-resolution and expensive equipment [4,5].

Optical fiber sensors (OFSs) can overcome these drawbacks as they allow for highly sensitive remote sensing, can be used in harsh environments and allow to produce chemical sensors and biosensors through the use of selective and active layers [6,7,8]. Plastic optical fibers (POFs) are a good option in comparison with glass optical fibers (GOFs) for the development of sensors as they are more flexible, easier to handle and manipulate, allowing low-cost sensing systems through the use of fibers with large diameters, which can be connected to low-precision and low-cost connectors. This brings the possibility of low-cost sensing systems based on intensity modulation [9,10].

The principle of operation of optical fiber chemical sensors and biosensors is usually based on the variations of the properties of the sensitive layer deposited on the fiber—commonly the refractive index (RI)—which occur due to the binding of a specific target (chemical species or family) to this layer and change the guiding characteristics of the light in the POF [11,12]. Light absorption [13,14,15] or emission such as fluorescence [16,17] are also optical principles which can be employed in chemical sensing or biosensing with POF. Cennamo et al. reported the development of several POF chemical sensors based on molecularly imprinted polymers (MIPs) deposited on a metal surface that covers the sensing region of a D-shaped POF [18]. Making use of surface plasmon resonance (SPR) and a wavelength-based optical configuration, the developed sensors show good performance with high sensitivity and low resolution, although expensive instruments for their characterization are required. In 2018, Cennamo et al. reported the development of a D-shaped POF chemical sensor based on intensity modulation [19], showing the reliability of this low-cost sensing platform to act as a chemical sensor for water quality assessment with the advantages of simpler, easier, and low-cost manufacturing procedures and experimental setup [20]. In this intensity-based configuration [19,20], the sensing region is much longer than the SPR D-shaped POF configuration [18]: 6 cm instead of 1 cm.

Most of the low-cost intensity-based POF-RI sensors reported in the literature refer to light attenuation as the sensing principle: absorption-based sensing, where light is absorbed by an absorbing medium; bending, as more light leaks out from the fiber, increasing the light loss and dependency on external refractive index; and evanescent field sensing which is enhanced by the improvement of the contact area between the light that travels in the POF and the external medium, such as by tapering, etching, polishing, or bending. Feng et al. reported in 2014 the development of a POF-RI sensor with a sensitivity of 950 µW/RIU and linear transmission loss (RI: 1.33–1.41), produced by double tapering (heat and pull method) and by decreasing the taper diameter to 200 µm [21]. In the same year, the depth of a side-polished POF and the curvature radius were also parameters studied by this group for refractive index sensing, where an increase in the transmission loss was also obtained with increasing RI (1.333–1.455) [22]. In 2015, Liu et al. reported a side-hole polished POF as a low-cost RI sensor, with a sensitivity of 1862.1 µW/RIU, which was dependent on the hole diameter. An increase in the sensor’s transmittance was obtained with the increase of the RI, from 1.34 to 1.475 [23]. The optimization of a U-bent unclad POF-RI sensor was reported by Gowri and Sai in 2016, which related the bending radius of the fiber for optimum sensitivity with the fiber diameter. In this case the transmission of light also decreased with the increase of the medium’s refractive index (1.33–1.37), and a resolution of 1 mRIU was obtained for measurements performed in terms of absorbance [24]. In 2017, Tiwari et al. reported a POF-RI sensor based on a spiral structure (~1.2 cm length, 300 µm depth, and 3 mm pitch of the spiral channel). The sensitivity of the sensor depended on the variation of the spiral pitch and applied strain, although the value of sensitivity was not presented [25]. Nevertheless, an increase in the output power measured in the detector was observed with the increase of the refractive index (around 1.34–1.41). In the same year, Teng et al. reported a U-bent side-polished POF-RI sensor in the range 1.33–1.44. The sensitivity to refractive index variation was improved by applying a curvature bending radius of 2 mm and a polished depth of 400 µm in a sensing region of 10 mm length [26]. With the U-bent configuration, the transmittance decreased with the increase of the refractive index, in opposition to the transmittance increase for straight configurations.

In the production of POF sensors for RI sensing there is a general concern about obtaining sensing regions with smooth surfaces [22,26], while roughness of the surface is generally not taken into account. Preliminary studies on the influence of surface roughness on the performance of D-shaped POF sensors for RI sensing were reported in 2016 by Sequeira et al. [27]. The final polishing procedure in the sensor’s production was performed with sandpapers of 5 µm and 1 µm grit size, revealing that a higher surface roughness leads to improved sensor performance—higher sensitivity and lower resolution. In 2018, Leal-Junior et al. reported a study where the roughness was included as an important parameter for the sensitivity of a POF curvature sensor based on intensity modulation, where the length, depth and curvature radius of the sensing region were also evaluated [28]. This study concluded that the roughness of the sensing region obtained with sandpapers P400 or P600 would allow to obtain a POF curvature sensor with better performance in terms of sensitivity, hysteresis and linearity. The principle of operation was based on light attenuation due to the bending of the POF, combined with the scattering losses due to the surface roughness. In 2019, Cennamo et al. presented a study on the effect of using different polishing papers—1 µm grit size, 5 µm grit size, or both—on the sensitivity of SPR D-shaped POF sensors [29].

Generally, the POF-RI sensors reported in the literature carry no information about the reproducibility of the manufacturing process, that is, the reproducibility of the characteristics of different sensors produced in the exact or a similar way. Furthermore, the repeatability of the sensor’s response in time is also mostly not addressed. Another important aspect is the fact that the majority of the reported studies do not take into account the variation of the transmitted light due to fluctuations of the light source, which can be overcome by referencing the obtained measurement.

Aiming future developments towards low-cost chemical sensors and biosensors, D-shaped POF sensors were produced and optimized for refractive index (RI) sensing with an optical setup based on the transmission of light through the POF, an LED as a light source and two photodiodes as detectors, allowing a self-referenced signal. At least three sets of experiments were performed for each sensor to verify the sensor’s repeatability, and five sensors were produced in similar way to verify the reproducibility of the manufacturing process.

The novelty of this study is to deepen the comprehension on the influence of the surface roughness in the performance of D-shaped POF-RI sensors by consecutive polishing with sandpapers of lower grit size without significantly changing the sensor thickness. The transmission losses due to the polishing procedures were evaluated, the morphology of the sensing region was analyzed with optical microscopy, and the D-shaped POF sensors were characterized with sucrose solutions of increasing refractive index. The developed sensors show good repeatability and the best performance was generally achieved after polishing with P600 sandpaper (higher sensitivity and lower resolution).

This study allows to better understand the light–matter interactions that contribute to the improvement of the sensing capabilities of low-cost POF sensors, produced by a simple and easy procedure and based on intensity modulation. Hopefully this will contribute to the future development of reliable, user-friendly, and low-cost POF chemical sensors.

## 2. Materials and Methods

Plastic Optical Fibers (POFs) commercially available from Asahi Kasei (DB-1000, Lot. No. E7221B 803) with a diameter of 1 mm (1000 ± 60 µm) and a step-index profile were selected. These POFs have the following characteristics: a numerical aperture of 0.5, a core of 980 µm made of poly(methyl methacrylate) (PMMA), and a fluorinated polymer cladding of 10 µm in thickness. The composition of the cladding was not made available by the manufacturer.

Five D-shaped POF sensors were produced by embedding POF samples of 20 cm in length in 3D-printed planar supports (6 cm long), followed by the polishing of the POF’s cladding and part of the core. Several polishing steps were performed using sandpapers of decreasing grit sizes. The D-shaped sensors were optically characterized after each polishing procedure in order to evaluate the dependency of the sensor’s performance on the roughness of the sensing region. The first polishing procedure was intended to remove the cladding and part of the core. The following polishing procedures were intended only to decrease the roughness of the sensing region without significantly changing its thickness.

A simple and low-cost optical configuration based on a transmission setup with a self-referenced signal allowed for characterization using one LED, one POF coupler and two photodiode detectors. The D-shaped POF sensors were characterized by the variation in the refractive index (RI) with sucrose solutions of increasing RI. Sucrose was commercially available in a common supermarket.

### 2.1. D-Shaped POF Sensors

**Figure 1:**
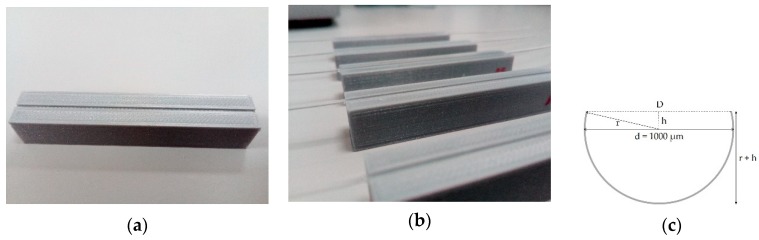
(**a**) Platform used for embedding the prepared fibers (groove dimensions: 6.0 cm, 1.1 mm, 0.7 mm); (**b**) produced D-shaped plastic optical fiber (POF) sensors (**c**) schematic representation of the D-shaped POF (d is the POF diameter, r the radius, and D the obtained surface thickness).

Several samples of fiber with 20 cm length were cut with a POF cutter, and the tips were polished in an “8-shaped” pattern with sandpapers of different grain sizes (5, 3, 1, 0.3 µm). The prepared samples were cleaned several times using distilled water and optical paper, after which they were embedded in grooves on 3D-printed planar supports with 6 cm length (see Figure 1a). The planar supports were made of Ultimaker PLA (polylactic acid) filament, printed with the Ultimaker 3 (instrumental error of 100 µm). The dimensions of the grooves were 6.0 cm length, 1.1 mm width, and 700 µm depth.

After gluing the fibers in the planar supports, the first polishing procedure (Polishing 1) was performed and five D-shaped POF sensors were produced—D1, D2, D3, D4, and D5 (see Figure 1b,c). Polishing 1 was performed manually until the platform was reached around 60 times with sandpaper P320 (~46 µm grit size) in an “8-shaped” pattern, after which the D-shaped sensors were washed several times with distilled water and cleaned with optical paper. The sensors were placed in the experimental setup and their performance was evaluated through the characterization with solutions of sucrose of known refractive index (RI).

The roughness of the sensing region was decreased by polishing with sandpapers of lower grit size: P600 (~26 µm, Polishing 2), 12 µm (Polishing 3) and 5 µm (Polishing 4).

All the polishing steps were made manually, with circular movements along the length of the sensors’ sensing region. After each polishing procedure the sensors were washed, and their performance for RI sensing was evaluated again through the characterization with sucrose solutions of increasing refractive index.

The surface of the sensing region was inspected via optical microscopy after each polishing procedure, and the average thickness with respective standard error was calculated.

**Figure 2:**
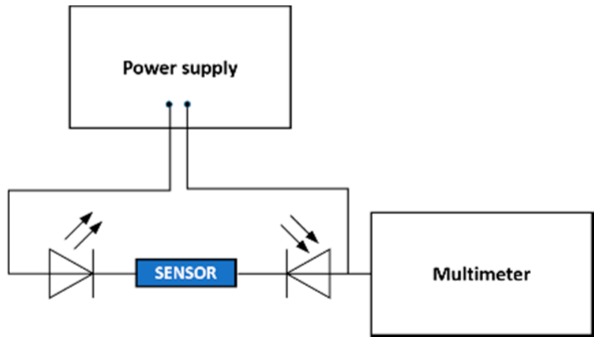
Schematic representation of the experimental setup used for the evaluation of the transmission losses due to the manufacturing procedures.

The transmission characteristics of the D-shaped POF sensors were evaluated in different steps of production. The end faces of the POFs were connected to an LED and a photodiode detector, which were then connected to a TTi bench power supply and a digital multimeter (see Figure 2). The output voltage was measured with the digital multimeter before and after the first polishing procedure (Polishing 1) and after the last polishing procedure and RI characterizations.

### 2.2. Refractive Index Sensing—Experimental Setup and Procedures

The performance of the D-shaped POF sensors for refractive index (RI) sensing was evaluated through characterization with sucrose solutions prepared in distilled water with RI varying from around 1.3326 (water) to 1.4118. The refractive indices of the prepared solutions (nD at 25°) were measured using a commercial refractometer (Abbemat 200, Anton Paar) with 1 × 10^−4^ resolution.

**Figure 3:**
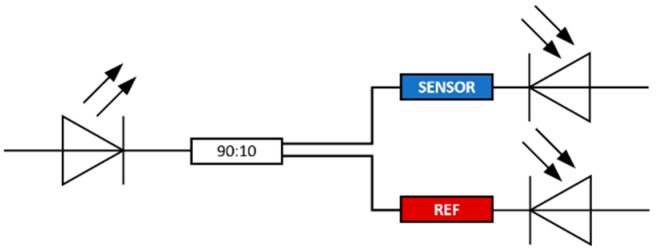
Schematic representation of the optical sensing setup.

An intensity-based transmission configuration was used for the sensors characterization: an LED (IF-E96, wavelength centred at 660 nm), a POF coupler (90:10, IF-542), and two photodiode detectors (IF-D91)—one directly connected to the D-shaped POF sensor and the other to the reference POF (see Figure 3). The data acquisition system contained an electronic plate that controlled the LED and the two photodiodes, a micro-processing unit that managed the data acquisition, a Bluetooth data transmitter and a battery.

The responsivity (r) of a photodiode can be defined as the ratio between the generated photocurrent ($I_{p}$) and the incident optical power (P) at a given wavelength (λ):

(1) $$r\left(\lambda \right)=\frac{I_{p}}{P}.$$

The received optical power can therefore be defined as

(2) $$P=\frac{I_{p}}{r\left(\lambda \right)}=\frac{V_{out}}{r\left(\lambda \right)\times R_{PD}},$$

where $V_{out}$ is the output voltage and $R_{PD}$ is the resistance of the photodetector.

The output voltages received at each photodetector ($V_{sensor}$ and $V_{ref}$) were monitored in real time with a LabVIEW application. The signal was self-referenced (k) to compensate for minor source fluctuations and external variations, calculated through the following equation (the responsivity is the same for both photodetectors):

(3) $$k=\frac{P_{sensor}}{P_{ref}}=\frac{V_{sensor}\times R_{PDref}}{R_{PDsensor}\times V_{ref}}.$$

**Figure 4:**
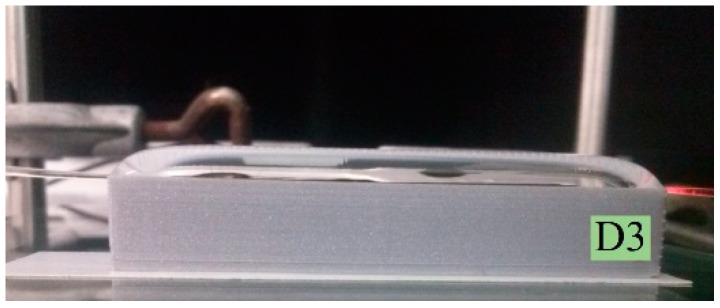
D-shaped POF sensor with the sensing region covered by water.

The response of the D-shaped POF sensors to the variation of RI was monitored in continuum, firstly recorded in distilled water ($k_{water}$) for 15 min, and after that the distilled water was removed and the next solution was added ($k_{solution}$) (see Figure 4). The sensing region was washed twice between measurements with the new solution in order to clean the fiber and platform from residues of the previous solution. The solutions were added and removed using plastic pipettes.

The transmitted signal normalized to water ($k_{norm}$) of the central 5 min of monitoring was calculated (average value and standard deviation) for each solution:

(4) $$k_{norm}=\frac{k_{solution}}{k_{water}}.$$

At least three replicated experiments (RI characterizations) were performed in order to verify the repeatability of the results and evaluate the performance of the sensors. The average values and respective standard deviations ($k_{avg}\pm \delta k_{avg}$) of the sensor responses were plotted against the average value and standard deviation of the measured RI of each solution, immediately after their removal from the sensors’ surface (measured with the commercial refractometer).

The sensitivity (S) and resolution (∆*n*) of the sensors were calculated as the variation of the normalized transmitted signal, the average value ($k_{avg}$), due to the refractive index (RI) variation:

(5) $$S=\frac{\partial k_{avg}}{\partial RI}$$

(6) $$∆n=\frac{1}{S}\times error_{max},$$

where $error_{max}$ corresponds to the maximum error obtained for each sensor in the set of experiments performed. The resolution is the minimum change in refractive index that can be detected.

## 3. Results and Discussion

The removal of the POF’s cladding and part of the core significantly reduces the quantity of light that travels through the fiber, reaching its end-face and the detector. This process allows for the development of low-cost sensors through very simple and cheap procedures, as it allows to increase the interaction with the external medium by changing the light propagation conditions.

In this study, several parameters were analyzed: losses due to the polishing procedures, the morphology of the sensing region and variation of the surface roughness, and their effects on the performance (sensitivity and resolution) of the D-shaped POF sensors in refractive index sensing.

### 3.1. D-Shaped POF Sensors—Losses Due to the Polishing Procedures

In order to assess the influence of the polishing procedures on the light losses, the samples of POF were connected to an LED (IF-E96) and a photodiode detector (IF-D91) and then connected to a TTi bench power supply ($V_{source}=1.50\;V$ and $I_{source}=1\;\mathrm{mA}$) and a digital multimeter ($V_{multimeter}$), as already described in Section 2.1.

Measurements were performed with air as the external medium (no liquid present in the POF sensing region). The output voltage was measured before and after embedding the POF samples in the planar support and after the first polishing procedure (measurements performed consecutively for all the prepared sensors).

The obtained results show that the transmission capacity of the optical fiber was not affected by the embedding process in the planar platform (light loss < −0.1%), although losses of light of around 90% to 94% were obtained after the first polishing (P320) (see Table 1). After all the polishing procedures and the sensors characterization with solutions of different refractive indices, the output voltage was again measured with the digital multimeter (performed consecutively for all the prepared sensors). The obtained results show that by decreasing the roughness of the sensing region (by polishing with sandpapers of decreasing grit size), the transmission of light through the POF can be increased even though more polishing was performed (see Table 1).

After the second polishing (P600), only a slight increase in the obtained output power was observed (light loss of around 92–93%). After the third polishing (12 µm grit size) the light loss was around 88–89%, and after the fourth (5 µm grit size) it was around 85–87%. These results show that the roughness of the sensing region is an important parameter in the transmission capacities of the POF.

Although a small variation was obtained between the measured output voltages for different sensors after each polishing procedure (from 1% to 4%), this is probably due to the manual manufacturing process. Before the polishing procedures, the measured output voltage was the same for all the samples.

### 3.2. D-Shaped POF Sensors—Morphology of the Sensing Region

The sensing region of the D-shaped POF sensors was evaluated via optical microscopy after each polishing procedure (see Table 2). The surface was observed using different magnifications and the thickness of the sensing region was calculated using the microscope’s software. Polishing the sensing region with sandpaper of a lower grit size decreases the roughness of the sensing region, easily observed by optical microscopy.

**Figure 5:**
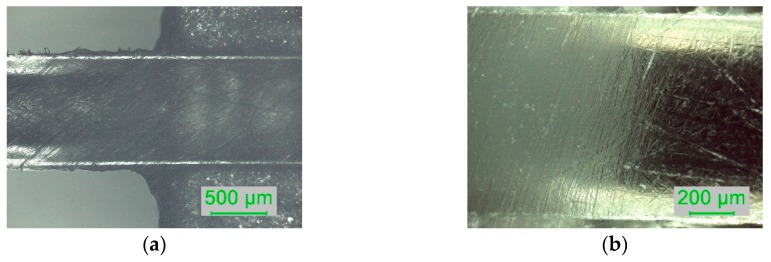
Optical microscopy images of the interface between the sensing region and the unpolished POF: (**a**) sensor D3 (P320, P600, 12 µm grit size); (**b**) close-up of sensor D1 (P320 and P600).

On the interface between the sensing region and the unpolished POF the roughness did not change in a well-defined way, revealing the difficulty of obtaining a clear interface between these two areas (depicted in Figure 5). This aspect can be improved. In fact, the D-shaped region should be limited to the zone immediately above the planar support and, outside of this region, the POF should be unpolished in order to prevent light losses that do not contribute to the sensing capabilities of the sensors. Furthermore, this aspect can be relevant for the sensors’ reproducibility, i.e., for achieving the same sensitivity in different sensors prepared in a similar way.

**Figure 6:**
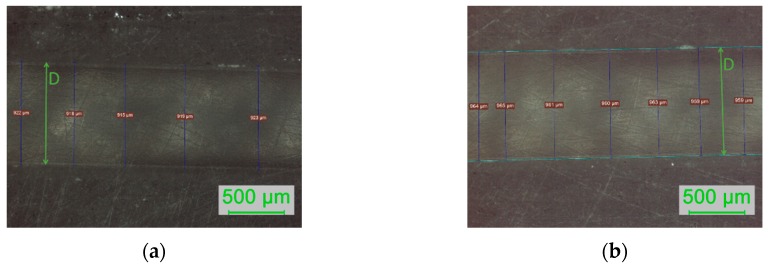
Optical microscopy images (in reflection, with a 5× objective lens): measurements of the thickness of the sensing region after Polishing 2 (P600) for sensor D2 (**a**) and sensor D3 (**b**).

The thickness of the sensing region was calculated as the average value and standard deviation of several measurements taken at different points on the surface, obtained from different optical microscopy images (see Table 3). As an example, in Figure 6 are depicted two images of optical microscopy with several performed measurements. Only images obtained with a 5× objective lens were used, as only this magnification allows to view completely the fiber diameter. After polishing with P320 sandpaper, the measurements were mostly not performed as it was very difficult to obtain clear images where the limits of the sensing region could be easily identified.

The differences obtained in the thickness of the produced sensors after polishing with the same sandpaper may be related to the manual process involved in the polishing procedure. Nevertheless, is worth pointing that the selected POF has a diameter error of 60 µm (manufacturer data), higher than the variation of thickness obtained. Furthermore, the length used for the measurements (microscope images) is only representative of the sensing region’s total length.

From the obtained thickness (D) it is possible to calculate the total height of the D-shaped sensors, 653 μm<(r+h)<748 μm, as determined by the Pythagorean theorem and considering the maximum thickness, d, as 1000 µm (see Figure 1c and Equation (7)).

(7) $$r^{2}=h^{2}+{\frac{D}{2}}^{2}$$

The total height obtained for the produced D-shaped POF sensors is in accordance with the groove depth on the planar supports (700 µm) with variations of around 50 µm.

### 3.3. Refractive Index Sensing

As described in Section 2.2, the response of the D-shaped POF sensors to the variation in refractive index (RI) was monitored in continuum.

**Figure 7:**
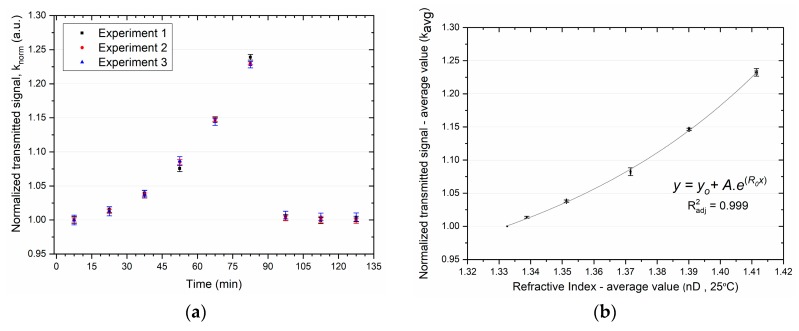
Response of the D-shaped POF sensor D2 when in contact with solutions of different refractive indices after Polishing 2 (P600): (**a**) normalized transmitted signal ($k_{norm}\pm \delta k_{norm}$) over time; (**b**) average value of the three replicated measurements ($k_{avg}\pm \delta k_{avg}$) and exponential fit.

As an example, the results obtained for three experiments with sensor D2 after polishing with sandpapers P320 and P600 are depicted in Figure 7. Figure 7a shows the normalized transmitted signal ($k_{norm}\pm \delta k_{norm}$) obtained with solutions of increasing refractive index and three washing steps with distilled water, revealing the reversibility of the sensors’ response. The average value and standard deviation were calculated ($k_{avg}\pm \delta k_{avg}$) and plotted against the average value and standard deviation of the measured refractive indices, depicted in Figure 7b. The repeatability was confirmed as the obtained relative error is 0.48% $\left(\frac{\delta k_{avg}{}_{max}}{k_{avg}{}_{max}}\times 100\right)$.

The best fitting applied to the obtained results is a nonlinear curve with an exponential model, as depicted in Figure 7b, with no weighting:

(8) $$y=y_{0}+A.e^{\left(R_{0}.x\right)}$$

**Figure 8:**
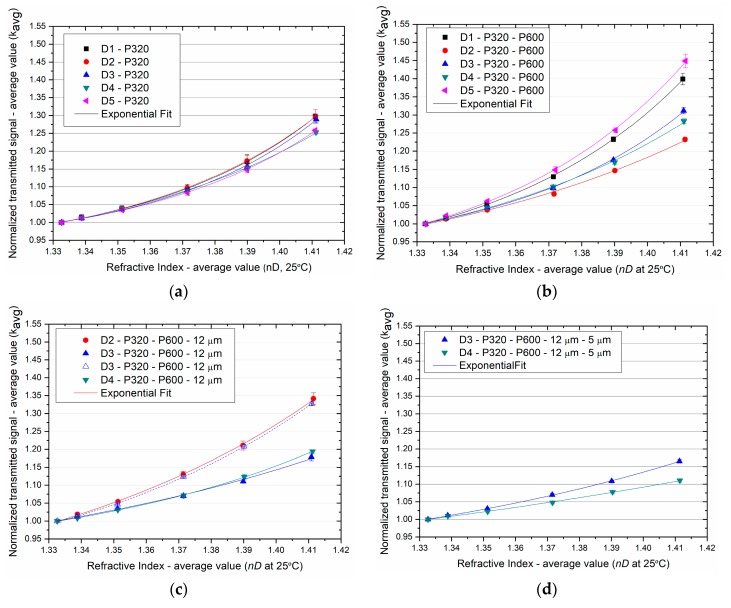
Variation of the normalized transmitted signal with increasing refractive index: (**a**) Polishing 1 (P320); (**b**) Polishing 2 (P600); (**c**) Polishing 3 (12 µm grit size); (**d**) Polishing 4 (5 µm grit size). Estimated grit size: P320 ~46 µm and P600 ~26 µm.

The sensor responses to RI variation after each polishing procedure are depicted in Figure 8 together with the exponential fitting of data. The error bars are the standard deviation of the set of measurements with each sensor and are related to the repeatability of the experiments ($\delta k_{avg}$). The fitting parameters and the maximum error obtained for each sensor (max(*δk_norm_*)) are listed in Table 4.

An increase in the transmitted signal through the D-shaped POF sensors was obtained with the increase of the external refractive index. After polishing with P320 sandpaper, an increase of around 25% to 30% in the light transmitted through the POF was observed (see Figure 8a). The response of the sensors was very similar until the refractive index reached around 1.37. With further increase of the refractive index, the sensors D1, D2, and D3 showed higher response.

After Polishing 2 (P600), depicted in Figure 8b, higher variation in the transmitted light (23% to 45%) with the same variation in refractive index was generally obtained. Contrary to the other sensors, sensor D2 showed a lower response ($k_{avg}\sim 1.23\;a.u.$ instead of ~1.29 a.u.) and initial transmitted light in water ($k_{water}=0.174\;a.u.$ instead of 0.179 a.u.). This can mean that this polishing procedure was not enough to change the guiding properties of the sensor D2 as the obtained results were very similar.

The D-shaped POF sensors D2, D3, and D4 were polished with a sandpaper of lower grit size (12 µm) and generally displayed a lower response (19% to 34% variation) (see Figure 8c). Two sets of experiments were performed with the sensor D3 and different responses were obtained. It was verified that the higher the initial transmitted signal ($k_{water}$), the higher the response obtained, revealing the importance of the connectorization between the POF sensor, the LED and the photodiode. A higher transmitted signal was expected with the decrease of the sensing region roughness, as discussed in Section 3.1. The lower transmitted signal firstly obtained for the sensor D3 can be related with a mismatch in the POF connectorization or dust in the fiber tip, and when the experiments were repeated, higher values were obtained. Nevertheless, after this polishing procedure, lower or similar response was obtained even with higher initial transmitted light in water.

After Polishing 4 (5 µm grit size) the variation in the transmitted light decreased with increasing RI for both sensors (variation from around 11% to 17%) (depicted in Figure 8d).

**Figure 9:**
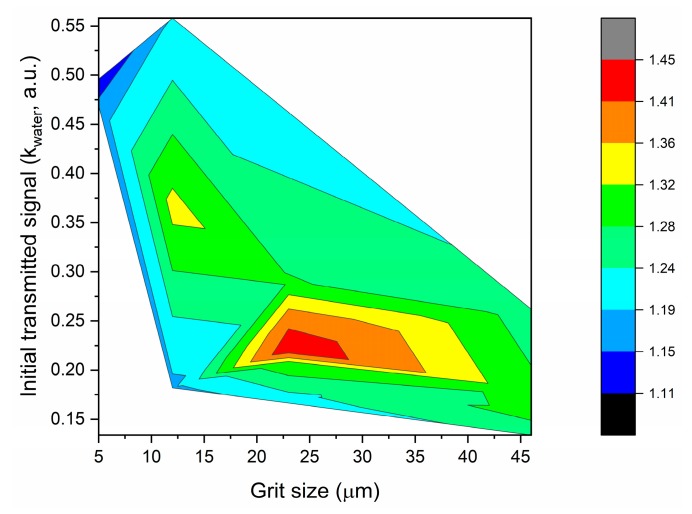
Maximum response of the D-shaped POF sensors (maximum value of *k_avg_*) with the initial transmitted signal ($k_{water}$) and the grit size of the polishing paper.

In order to better understand the influence of the initial transmitted light ($k_{water}$) and the surface roughness in the sensor’s signal variation (maximum value of $k_{avg}$), all the data were analyzed together, as depicted in Figure 9.

From the analysis of the light losses in the POF sensors with the polishing procedures (Section 3.1), it was observed that by polishing with a sandpaper of lower grit size (leading to lower surface roughness) the scattering of the light decreases, increasing the transmitted light through the POF sensor (higher output voltage measured in the photodiode).

It was verified that lower surface roughness (obtained after polishing with finer sandpaper with grit sizes 12 µm or 5 µm) generally resulted in higher light transmission but lower sensor response to RI variation (Figure 9). By polishing with coarser sandpapers, e.g., P320 with grit size of ~46 µm, the response of the sensors was similar to when the sandpaper of 12 µm grit size was used, even if a decrease in the initial transmitted signal was obtained. In summary, the best sensor performance was achieved when a balance between roughness (enhancing the interaction of light and the external medium) and transmission losses due to polishing was obtained. The results show that this balance was achieved when the sensors were polished with P600 sandpaper (~26 µm grit size).

It was also observed that the response of the sensors that were polished with sandpapers of lower grit size was more linear (lower values of $R_{0}$). For that reason, sensor responses were also fitted using linear regression and the obtained values of the adjusted R-squared are listed in Table 5.

Clearly, the linearity of the response of the D-shaped POF sensors increases with the decreasing roughness of the sensing region. Nevertheless, linearity is not mandatory in sensing, the sensors can be characterized and calibrated as they give a repeatable and recoverable response, as verified.

The exponential fit applied to the obtained results allowed to calculate the sensitivity and the resolution of the sensors according to Equations (5), (6), and (8):

(9) $$S=\frac{\partial k_{avg}}{\partial RI}=R_{0}.A.e^{\left(R_{0}.RI\right)}$$

(10) $$∆n=\frac{1}{S}\times error_{max}=\frac{1}{R_{0}.A.e^{\left(R_{0}.RI\right)}}.\max\left(\delta k_{norm}\right).$$

**Figure 10:**
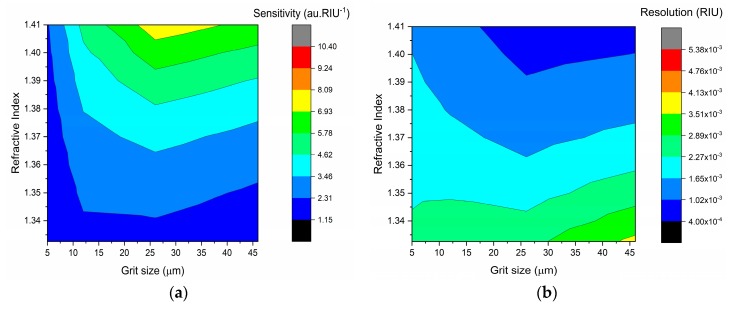
Sensitivity (**a**) and resolution (**b**) of the D-shaped POF sensors.

The performance of the sensors is not only dependent on the increase of the transmitted light with refractive index (sensitivity) but also on the maximum error obtained for each sensor, which is used to calculate the resolution of the sensors. The sensitivity and resolution calculated according to Equations (9) and (10) are depicted in Figure 10.

The sensitivity and resolution of the D-shaped POF sensors depend on the external RI: the higher the refractive index of the external medium, the higher the sensitivity and lower the resolution of the sensors. The sensors performance is also dependent on the roughness of the sensing region. In general, the sensors showed higher sensitivities and lower resolutions after Polishing 2 (P600) (see Figure 10). A resolution of 10^−4^ RIU was achieved for values of RI higher than 1.36 for sensor D5, 1.37 for sensor D1, 1.38 for sensor D4, and 1.40 for sensor D3 after polishing with P600 sandpaper; for the sensor D2, a resolution of 10^−4^ RIU was obtained for RI higher than 1.36 after Polishing 3 (12 µm grit size).

Polishing with coarser sandpaper leads to higher roughness of the sensing region and, consequently, higher scattering of the light and less light transmission through the fiber, as already discussed and validated. With the increase of the refractive index of the external medium, an increase in the transmitted light through the POF is observed, independently of the roughness of the sensing region. Considering the external medium as a substitute cladding, a decrease in the transmitted light would be expected with the increase of the refractive index, as at higher external RI, the angle needed for the total internal reflection (TIR) to occur also would be higher. According to Snell’s law of refraction, for the RI variation from 1.3326 (water) to 1.41, the critical angle would increase from around 63° to 71°, which means that fewer light rays would satisfy the condition for TIR, and, consequently, more light would be refracted and less light would be totally transmitted, reaching the detector. However, this is true considering smooth flat surfaces, while for rough surfaces scattering will play an important role. As the obtained results show, an increase in the medium refractive index leads to a decrease in the scattering losses caused by the roughness of the sensing region, as a higher refractive index will allow for the surface to appear smoother to the light ray that travels in the POF, resulting in the observed increase in transmitted light.

Lower surface roughness means lower scattering and higher transmitted light; however, at the same time, it also means that less light will interact with the external medium and the same variation in the refractive index will cause a lower variation in the transmitted signal, resulting in the increasingly linear response with decreasing surface roughness. In opposition, rough surfaces mean higher scattering and less light transmitted through the POF but also higher interaction with the external medium with an exponential response to RI variations. Therefore, a balance in surface roughness is needed in order to optimize the sensors performance.

In the studies reported in the literature, P600 was also found to be the polishing paper that allowed better sensor performance in terms of sensitivity to be obtained [28]. In this case, an increase in linearity was not observed after polishing with this sandpaper. On the contrary, it was observed that smoother surfaces allow for more linearity in the sensor response. Furthermore, when several polishing papers are used in the sensor’s manufacture, care should be taken as the previous polishing procedure can affect and influence the sensor response if the surface roughness is not properly changed by the new polishing procedure, as reported in [29].

## 4. Conclusions

The roughness of the sensing region is an important parameter for the transmission capacities of POFs and, consequently, for their sensing performance. Smoother surfaces allow for more light being transmitted, whereas rougher surfaces lead to more scattering losses and, therefore, less light transmission through the POF. At the same time, correct adjustment of the surface roughness allows to increase the sensor response to changes in the external medium properties, such as refractive index variations.

Despite the manual manufacturing process of these sensors, it was verified that the thickness of the sensing region and the consequent height of the D-shaped sensors were very similar, with a variation smaller than the thickness variation given by the POF manufacturer. Also, the roughness of the sensing region can be directly controlled by using sandpaper with specific grain sizes.

In general, the best performances were achieved after polishing the sensing region with P600 sandpaper, and by smoothing the surface lower sensitivity and higher resolution were obtained. Smoother surfaces allow higher linearity in the sensor response, although this is not an important request as sensors with nonlinear response can be used as long as they are repeatable and reversible. A resolution of 10^−3^–10^−4^ RIU was obtained, depending on the value of the external refractive index.

The reproducibility of the sensors was verified after the first polishing procedure (P320) as a similar response was obtained with increasing RI. Less reproducible behavior was observed after the following polishing procedures, which may be due to the manual manufacturing process resulting in an irregular interface between the unpolished POF and the sensing region. This aspect should be improved in the future in order to obtain higher reproducibility and to avoid scattering losses that do not contribute to the sensing capabilities of the sensors. Furthermore, the connectorization between the POFs and the LED and photodiodes is also an important parameter that can affect the reproducibility.

The lack of normalization in the calculation of the performance of POF-RI sensors makes it difficult to compare the obtained results with those reported in the literature. Nevertheless, the obtained resolution was similar to that reported in [24] (1 mRIU), although it was dependent on the external refractive index (10^−3^–10^−4^ RIU). The obtained sensitivity and resolution suggest that these sensors are suitable for chemical sensing developments, as already reported in [19].

The surface roughness of the sensing region is of extreme importance when developing POF chemical sensors, as the variation in the refractive index that occurs in the selective receptor layer through the binding of the target analyte will allow for chemical detection. The developed optical sensing system allows for low-cost, on-site and remote sensing, paving the way for cheaper, simpler and reliable sensing tools for water quality assessment.

## Figures and Tables

**Table 1:** Calculated light losses (%) in different production stages of the D-shaped POF sensors (relative to the unpolished POF sample).

Light Loss (%)					
Sensors	Unpolished POF	D-Shaped POF (Polished, Grit Size)			
	Embedded	P320 (~46 µm)	P600 (~26 µm)	12 µm	5 µm
**D1**	<−0.1	93.8	93.3		
**D2**		93.3	-	(88.8–88.2)	
**D3**		94.4	-	-	(87.1–86.6)
**D4**		90.5	-	-	(86.0–85.4)
**D5**		(93.3–92.7)	92.2		

The negative sign “−” means that the transmitted light measured in the terminals of the POF increased.

**Table 2:** D-shaped POF sensors: optical microscopy images of the sensing region after each polishing procedure (in reflection, with a 10× objective lens).

Sensors	Polishing Procedures (Sandpaper Grit Size)			
	P320 (~46 µm)	P600 (~26 µm)	12 µm	5 µm
**D1**	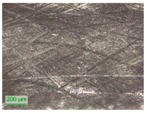	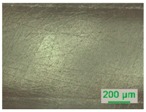		
**D2**	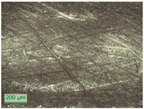	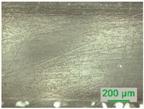	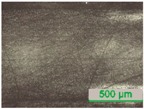	
**D3**	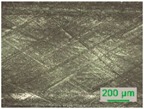	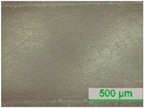	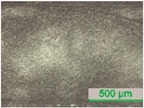	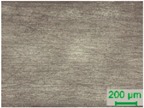
**D4**	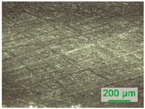	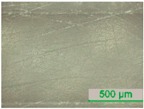	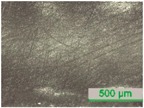	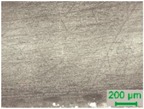
**D5**	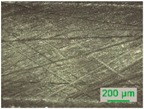	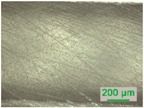		

**Table 3:** D-shaped POF sensors: thickness of the sensing region after the polishing procedures, average value and standard deviation (*n* is the number of measurements performed).

Sensor	Thickness of the Sensing Region (*D*, µm)			
	P320 (~46 µm)	P600 (~26 µm)	12 µm	5 µm
**D1**	x	875 ± 12 (*n* = 33)	--	--
**D2**	x	912 ± 07 (*n* = 29)	896 ± 05 (*n* = 14)	--
**D3**	934 ± 08 (*n* = 13)	952 ± 10 (*n* = 14)	947 ± 10 (*n* = 11)	944 ± 06 (*n* = 12)
**D4**	x	888 ± 08 (*n* = 13)	893 ± 11 (*n* = 14)	868 ± 06 (*n* = 20)
**D5**	x	936 ± 19 (*n* = 13)	--	--

**Table 4:** Obtained results from the RI characterizations and exponential fit.

Sensor	max(*δk_norm_*) (a.u.)	*R*_0_	*A*	Reduced *χ*^2^	Adj. *R*^2^
**Polishing 1—P320 (~46 µm)**					
**D1**	5.67 × 10^−3^	19.7380 ± 0.9183	(3.0147 ± 3.9643) × 10^−13^	5.7387 × 10^−6^	0.99956
**D2**	5.67 × 10^−3^	17.6686 ± 0.9552	(5.8028 ± 7.9641) × 10^−12^	6.2849 × 10^−6^	0.99951
**D3**	5.23 × 10^−3^	20.8698 ± 1.3336	(0.5741 ± 1.0948) × 10^−13^	1.1250 × 10^−5^	0.99908
**D4**	3.86 × 10^−3^	15.5884 ± 0.6314	(9.9297 ± 9.0497) × 10^−11^	2.0756 × 10^−6^	0.99978
**D5**	7.54 × 10^−3^	19.1898 ± 0.6959	(5.7353 ± 5.7199) × 10^−13^	2.5028 × 10^−6^	0.99974
**Polishing 2—P320 (~46 µm)—P600 (~26 µm)**					
**D1**	4.61 × 10^−3^	18.1293 ± 0.6274	(4.0916 ± 3.6850) × 10^−12^	4.9376 × 10^−6^	0.99979
**D2**	7.07 × 10^−3^	12.9652 ± 1.3225	(4.0754 ± 7.8431) × 10^−9^	8.0058 × 10^−6^	0.99900
**D3**	5.94 × 10^−3^	19.5214 ± 1.2709	(4.2817± 7.7950) × 10^−13^	1.1990 × 10^−5^	0.99915
**D4**	3.72 × 10^−3^	15.4351± 1.1793	(1.3817± 2.3529) × 10^−10^	8.9359 × 10^−6^	0.99923
**D5**	4.25 × 10^−3^	18.4696 ± 0.8383	(2.7554 ± 3.3151) × 10^−12^	1.1189 × 10^−5^	0.99962
**Polishing 3—P320 (~46 µm)—P600 (~26 µm)—12 µm**					
**D2**	3.44 × 10^−3^	12.1647 ± 0.6685	(1.9225 ± 1.8762) × 10^−8^	4.4856 × 10^−6^	0.99974
**D3**	6.48 × 10^−3^	8.1991 ± 3.0627	(0.3456 ± 1.5845) × 10^−5^	2.4399 × 10^−5^	0.99461
	7.32 × 10^−3^	12.0327 ± 0.9656	(2.2928 ± 3.2333) × 10^−8^	8.6735 × 10^−6^	0.99947
**D4**	3.77 × 10^−3^	12.3319 ± 0.6847	(8.6758 ± 8.6656) × 10^−9^	1.5171 × 10^−6^	0.99973
**Polishing 4—P320 (~46 µm)—P600 (~26 µm)—12 µm—5 µm**					
**D3**	3.19 × 10^−3^	8.0865 ± 0.6820	(3.8485 ± 3.9345) × 10^−6^	1.1013 × 10^−6^	0.99973
**D4**	3.19 × 10^−3^	4.0643 ± 1.8767	(1.2900 ± 3.9300) × 10^−3^	3.7801 × 10^−6^	0.99794

**Table 5:** Obtained adjusted R-squared values from linear regression.

Sensor	Sandpaper			
	P320 (~46 µm)	P600 (~26 µm)	12 µm Grit Size	5 µm Grit Size
**D1**	0.94575	0.95417	-	-
**D2**	0.95513	0.97251	0.97823	-
**D3**	0.94009	0.94718	0.97839–0.97865	0.99037
**D4**	0.96572	0.96648	0.97791	0.99368
**D5**	0.94895	0.95200	-	-

## References

[1] Le T.D.H., Scharmüller A., Kattwinkel M., Kühne R., Schüürmann G., Schäfer R.B. (2017). Contribution of waste water treatment plants to pesticide toxicity in agriculture catchments. Ecotoxicol. Environ. Saf..

[2] Garcia-Ivars J., Martella L., Massella M., Carbonell-Alcaina C., Alcaina-Miranda M.I., Iborra-Clar M.I. (2017). Nanofiltration as tertiary treatment method for removing trace pharmaceutically active compounds in wastewater from wastewater treatment plants. Water Res..

[3] Li N., Sheng G.P., Lu Y.Z., Zeng R.J., Yu H.Q. (2017). Removal of antibiotic resistance genes from wastewater treatment plant effluent by coagulation. Water Res..

[4] Gholizadeh M.H., Melesse A.M., Reddi L. (2016). A Comprehensive Review on Water Quality Parameters Estimation Using Remote Sensing Techniques. Sensors.

[5] Richardson S.D., Ternes T.A. (2018). Water Analysis: Emerging Contaminants and Current Issues. Anal. Chem..

[6] Wang X.-D., Wolfbeis O.S. (2016). Fiber-Optic Chemical Sensors and Biosensors (2013–2015). Anal. Chem..

[7] Pospíšilová M., Kuncová G., Trögl J. (2015). Fiber-optic chemical sensors and fiber-optic bio-sensors. Sensors.

[8] Elosua C., Arregui F.J., Villar I.D., Ruiz-Zamarreño C., Corres J.M., Bariain C., Goicoechea J., Hernaez M., Rivero P.J., Socorro A.B. (2017). Micro and Nanostructured Materials for the Development of Optical Fibre Sensors. Sensors.

[9] Bilro L., Alberto N., Pinto J.L., Nogueira R. (2012). Optical sensors based on plastic fibers. Sensors.

[10] Jin Y., Granville A.M. (2016). Polymer Fiber Optic Sensors—A Mini Review of their Synthesis and Applications. Biosens. Bioelectron..

[11] Cennamo N., Testa G., Marchetti S., De Maria L., Bernini R., Zeni L., Pesavento M. (2017). Intensity-based plastic optical fiber sensor with molecularly imprinted polymer sensitive layer. Sens. Actuators B Chem..

[12] Lopes R.N., Rodrigues D.M.C., Allil R.C.S.B., Werneck M.M. (2018). Plastic optical fiber immunosensor for fast detection of sulfate-reducing bacteria. Meas. J. Int. Meas. Confed..

[13] Foguel M.V., Ton X.A., Zanoni M.V.B., Sotomayor M.D.P.T., Haupt K., Bui B.T.S. (2015). A molecularly imprinted polymer-based evanescent wave fiber optic sensor for the detection of basic red 9 dye. Sens. Actuators B Chem..

[14] Khalaf A.L., Mohamad F.S., Rahman N.A., Lim H.N., Paiman S., Yusof N.A., Mahdi M.A., Yaacob M.H. (2017). Room temperature ammonia sensor using side-polished optical fiber coated with graphene/polyaniline nanocomposite. Opt. Mater. Express.

[15] Azkune M., Ruiz-Rubio L., Aldabaldetreku G., Arrospide E., Pérez-Álvarez L., Bikandi I., Zubia J., Vilas-Vilela J. (2018). U-Shaped and Surface Functionalized Polymer Optical Fiber Probe for Glucose Detection. Sensors.

[16] Rivera L., Izquierdo D., Garcés I., Salinas I., Alonso J., Puyol M. (2009). Simple dip-probe fluorescence setup sensor for in situ environmental determinations. Sens. Actuators B Chem..

[17] Ton X.A., Acha V., Bonomi P., Bui B.T.S., Haupt K. (2015). A disposable evanescent wave fiber optic sensor coated with a molecularly imprinted polymer as a selective fluorescence probe. Biosens. Bioelectron..

[18] Cennamo N., D’Agostino G., Porto G., Biasiolo A., Perri C., Arcadio F., Zeni L. (2018). A molecularly imprinted polymer on a plasmonic plastic optical fiber to detect perfluorinated compounds in water. Sensors.

[19] Cennamo N., D’Agostino G., Sequeira F., Mattiello F., Porto G., Biasiolo A., Nogueira R., Bilro L., Zeni L. (2018). A simple and low-cost optical fiber intensity-based configuration for perfluorinated compounds in water solution. Sensors.

[20] Sequeira F., Duarte D., Bilro L., Rudnitskaya A., Pesavento M., Zeni L., Cennamo N. (2016). Refractive Index Sensing with D-Shaped Plastic Optical Fibers for Chemical and Biochemical Applications. Sensors.

[21] Feng D.-J., Liu G., Liu X., Jiang M., Sui Q. (2014). Refractive index sensor based on plastic optical fiber with tapered structure. Appl. Opt..

[22] Feng D.-J., Zhang M.-S., Liu G., Liu X.-L., Dong-Fang J. (2014). D-Shaped Plastic Optical Fiber Sensor for Testing Refractive Index. IEEE Sens..

[23] Liu G., Feng D., Zhang M., Jiang S., Ye Z. (2015). Side-Hole Plastic Optical Fiber for Testing Liquid’s Refractive Index. IEEE Sens. J..

[24] Gowri A., Sai V.V.R. (2016). Development of LSPR based U-bent plastic optical fiber sensors. Sens. Actuators B Chem..

[25] Tiwari S., Singh M.K., Pandey P.C. (2017). Refractive index sensor based on a multi-notched plastic optical fiber. IEEE Sens. J..

[26] Teng C., Yu F., Jing N., Ding Y., Si Z., Zheng J. (2017). Investigation of refractive index sensors based on side-polished plastic optical fibers. Opt. Fiber Technol..

[27] Sequeira F., Duarte D., Nogueira R., Rudnitskaya A., Cennamo N., Zeni L., Bilro L. Analysis of the roughness in a sensing region on D-shaped POFs. Proceedings of the 25th International Conference on Plastic Optical Fibers.

[28] Leal-Junior A.G., Frizera A., Pontes M.J. (2018). Sensitive zone parameters and curvature radius evaluation for polymer optical fiber curvature sensors. Opt. Laser Technol..

[29] Cennamo N., Pesavento M., Marchetti S., de Maria L., Zuppella P., Zeni L. (2019). Polishing Process Analysis for Surface Plasmon Resonance Sensors in D-Shaped Plastic Optical Fibers. Sensors. CNS 2018. Lecture Notes in Electrical Engineering.

